# In the national epidemiological bulletins – a selection from recent issues

**DOI:** 10.2807/1560-7917.ES.2018.23.39.1809271

**Published:** 2018-09-27

**Authors:** Eurosurveillance editorial team

**Affiliations:** 1 European Centre for Disease Prevention and Control (ECDC), Stockholm, Sweden

**Keywords:** infectious diseases, public health, Europe

**Healthcare-associated infections and antibiotic resistance**

CPO guideline and mandatory notification

EPI-NEWS 37/2018

12 September 2018, Denmark

https://www.ssi.dk/English/News/EPI-NEWS/2018/No%2037%20-%202018.aspx

 

*Bacillus cereus* bacteremia in neonatal resuscitation at AP-HP (FRANCE) in 2016

Bulletin épidémiologique hebdomadaire, 25-26/2018

17 July 2018, France

http://invs.santepubliquefrance.fr/beh/2018/25-26/pdf/2018_25-26.pdf

 

**Food- and waterborne diseases**

Salmonella in the Netherlands in 2017

Infectieziekten Bulletin 2018; 29(7)

September 2018, the Netherlands

https://magazines.rivm.nl/2018/09/infectieziekten-bulletin/salmonella-nederland-2017

 

Marine bacteria in bathing water

EPI-NEWS 28-33/2018

15 August 2018, Denmark

https://www.ssi.dk/English/News/EPI-NEWS/2018/No%2028-33%20-%202018.aspx

 

Botulism outbreak in Southern Jutland

EPI-NEWS 25/2018

20 June 2018, Denmark

https://www.ssi.dk/English/News/EPI-NEWS/2018/No%2025%20-%202018.aspx

 

**Hepatitides**

Hepatitis E surveillance in France, 2002-2016

Bulletin épidémiologique hebdomadaire, 28/2018

11 September 2018, France

http://invs.santepubliquefrance.fr/beh/2018/28/pdf/2018_28.pdf

 

Laboratory reports of hepatitis A infections in England and Wales, 2017Health Protection Report; 12(27)

27 July 2018, United Kingdom

https://assets.publishing.service.gov.uk/government/uploads/system/uploads/attachment_data/file/730084/hpr2718_hav-nnl.pdf

 

Viral hepatitis B and D in 2017

Epidemiologisches Bulletin 30/2018

26 July 2018, Germany

https://www.rki.de/DE/Content/Infekt/EpidBull/Archiv/2018/Ausgaben/30_18.pdf?__blob=publicationFile

 

Hepatitis C: situation in Germany in 2017

Epidemiologisches Bulletin 29/2018

19 July 2018, Germany

https://www.rki.de/DE/Content/Infekt/EpidBull/Archiv/2018/Ausgaben/29_18.pdf?__blob=publicationFile

 

Hepatitis C in Ireland 2018

Epi-Insight 2018;19(7)

July 2018, Ireland

http://ndsc.newsweaver.ie/epiinsight/g5q8i4gp2v8n0ykqgxo0gg?a=1&p=53582193&t=17517774

 

**Vaccine-preventable diseases**

 

Measles outbreaks in Ireland and Europe - updated NIAC guidance on pre travel vaccination

Epi-Insight 2018;19(9)

September 2018, Ireland

http://ndsc.newsweaver.ie/epiinsight/1lemwohj4zln0ykqgxo0gg?a=1&p=53827184&t=17517774

 

Current epidemiology of measles in Germany

Epidemiologisches Bulletin 33/2018

16 August 2018, Germany

https://www.rki.de/DE/Content/Infekt/EpidBull/Archiv/2018/Ausgaben/33_18.pdf?__blob=publicationFile

 

Influenza and pertussis vaccine uptake in pregnant women, Ireland 2017-2018

Epi-Insight 2018;19(8)

August 2018, Ireland

http://ndsc.newsweaver.ie/epiinsight/1uau3bfmrmin0ykqgxo0gg?email=true&a=2&p=53699216&t=17517804

 

**Respiratory diseases**

 

Vaccination coverage and willingness to get vaccinated among hospital staff during the influenza season 2016/17

Epidemiologisches Bulletin 32/2018

9 August 2018, Germany

https://www.rki.de/DE/Content/Infekt/EpidBull/Archiv/2018/Ausgaben/32_18.pdf?__blob=publicationFile

 

Outbreaks of acute respiratory infections in nursing homes based in Pays de la Loire (France). From reporting to surveillance

Bulletin épidémiologique hebdomadaire, 25-26/2018

17 July 2018, France

http://invs.santepubliquefrance.fr/beh/2018/25-26/pdf/2018_25-26.pdf

 

Legionellosis in the spotlight

Epi-Insight 2018;19(7)

July 2018, Ireland

http://ndsc.newsweaver.ie/epiinsight/1trs1jhg5yyn0ykqgxo0gg?email=true&a=2&p=53582186&t=17517804

 

**Sexually transmitted diseases**

Pre-exposure prophylaxis awareness and use among men who have sex with men attending gay venues in five French cities. Prevagay 2015

Bulletin épidémiologique hebdomadaire, 29/2018

25 September 2018, France

http://invs.santepubliquefrance.fr/beh/2018/29/pdf/2018_29.pdf

 

Imported case of ceftriaxone resistant gonorrhoea in Ireland, 2018

Epi-Insight 2018;19(9)

September 2018, Ireland

http://ndsc.newsweaver.ie/epiinsight/1nwigkkpz7zn0ykqgxo0gg?email=true&a=2&p=53827179&t=17517804

 

Unlinked anonymous HIV and viral hepatitis monitoring among PWID: 2018 report

Health Protection Report; 12(27)

27 July 2018, United Kingdom

https://assets.publishing.service.gov.uk/government/uploads/system/uploads/attachment_data/file/729614/hpr2718_uam-pwid.pdf

 

**Zoonoses and vector-borne diseases**

Seoul virus in the Netherlands

Infectieziekten Bulletin 2018; 29(7)

September 2018, the Netherlands

https://magazines.rivm.nl/2018/09/infectieziekten-bulletin/seoulvirus-nederland

 

Chikungunya, dengue and Zika virus infection surveillance in mainland France, 2017

Bulletin épidémiologique hebdomadaire, 24/2018

10 July 2018, France

http://invs.santepubliquefrance.fr/beh/2018/24/pdf/2018_24.pdf

 

Autochthonous chikungunya circulation in two municipalities from the Var department (France), August-September 2017 

Bulletin épidémiologique hebdomadaire, 24/2018

10 July 2018, France

http://invs.santepubliquefrance.fr/beh/2018/24/pdf/2018_24.pdf

 

Public perceptions and prevention behaviours related to arboviral diseases in Metropolitan France: 2016 Health Barometer

Bulletin épidémiologique hebdomadaire, 24/2018

10 July 2018, France

http://invs.santepubliquefrance.fr/beh/2018/24/pdf/2018_24.pdf

 

Imported malaria 2017

EPI-NEWS 26/2018

27 June 2018, Denmark

https://www.ssi.dk/English/News/EPI-NEWS/2018/No%2026%20-%202018.aspx

 

**Other**

Screening of pregnant women for hepatitis B, HIV and syphilis, 2017

EPI-NEWS 36/2018

5 September 2018, Denmark

https://www.ssi.dk/English/News/EPI-NEWS/2018/No%2036%20-%202018.aspx

